# Optimized Extraction of Polysaccharides from *Grateloupia* *livida* (Harv.) Yamada and Biological Activities

**DOI:** 10.3390/molecules200916817

**Published:** 2015-09-16

**Authors:** Danyan Ye, Zebin Jiang, Fuchun Zheng, Hongmei Wang, Yanmei Zhang, Fenfei Gao, Peihong Chen, Yicun Chen, Ganggang Shi

**Affiliations:** 1 Department of Pharmacology, Shantou University Medical College, Shantou 515041, China; E-Mails: 15917907394@163.com (D.Y.); junebulu@163.com (H.W.); ymzhang@stu.edu.cn (Y.Z.); ffgao@stu.edu.cn (F.G.); m13411961378@163.com (P.C.); 2 Department of Pharmacy, First Affiliated Hospital, Shantou University Medical College, Shantou 515041, China; E-Mails: jzbwood@126.com (Z.J.); zhengfuchunsh@163.com (F.Z.); 3 Traditional Chinese Medicine Laboratory, Shantou University Medical College, Shantou 515041, China; 4 Department of Cardiovascular Diseases, First Affiliated Hospital, Shantou University Medical College, Shantou 515041, China

**Keywords:** *Grateloupia livida* (Harv.) Yamada, polysaccharide extraction, Box-Behnken design, antioxidant and anti-cancer activity

## Abstract

Polysaccharides from *Grateloupia* *livida* (Harv.) Yamada (GL) were extracted by a heating circumfluence method. Single-factor experiments were performed for the three parameters: extraction time (X_1_), extraction temperature (X_2_) and the ratio of water to raw material (X_3_) and their test range. From preliminary experimental results, one type of the response surface methodology, the Box-Behnken design was applied for the optimizing polysaccharide extraction conditions. The experimental data obtained were fitted to a second-order polynomial equation. The optimal conditions were extraction time 5 h, extraction temperature 100 °C and ratio of water to raw material 70 mL/g. Under these conditions, the experimental yield was 39.22% ± 0.09%, which well matched the predicted value (39.25%), with 0.9774 coefficient of determination (*R*^2^). GL polysaccharides had scavenging activities for DPPH and hydroxyl radicals *in vitro*. The scavenging rates for both radicals peaked at 20 mg/mL GL concentration. However, the positive standard, VC (ascorbic acid), possessed stronger antioxidant activities than GL polysaccharides. Furthermore, the anticancer activity of GL polysaccharides on HepG2 cell proliferation increased dose- and time-dependently, but the positive standard, 5-fluorouracil (5-fu, showed more significant anticancer activity in this study. Overall, GL polysaccharides may have potential applications in the medical and food industries.

## 1. Introduction

Marine algae are classified by their colors, mainly red, green, and brown algae. The red algae have a special red color because of the algae pigments and accessory pigments, and different living water layers lead to different ratios of accessory pigments, so different species of red algae have different colors, ranging from yellowish-red to purplish-red [1]. Worldwide, red algae represent 4000 species in 500 genera: the most common species are *Gloiopeltis tenax* (Turn.) J. Ag, *Gracilaria verrucosa* (Huds.) Papentf, *Grateloupia filicina.* C. Ag, *Grateloupia livida* (Harv.) Yamada and *Grateloupia* *turuturu* [2]. In recent years, most research has involved the Grateloupia family, finding that extracts from the Grateloupia family have wide biological effects such as antiangiogenic [3], antioxidant [4], antiviral [5], anti-allergic [6], and anticancer activities [7].

*G. livida* (Harv.) Yamada (GL), a red algae belonging to Rhodophyta, Rhodophyceae, Gigartinales, Halymeniaceae, is mainly distributed in the coastal warm temperate zone [8]. It is an edible and medicinal seaweed, usually used for treating sore throat, stomachache, ascariasis and seaworm infections and dysentery [9]. Red algae (Rhodophyta) are characterized by their content of non-fibrillar and sulfated polysaccharides such as carrageenans, agars and complex sulfated galactans, which are classified by their structural features [10,11]. Sulfated polysaccharides are acidic polysaccharides containing sulfate groups of natural and semi-synthetic anionic compounds [12]. GL polysaccharides are mainly composed of galactose linked with sulfate ester, and the exquisite structure including the replacement mode of sulfate ester is related to its bioactivities [13]. Many studies were performed to demonstrate that polysaccharides from the Grateloupia family had outstanding antioxidant and anticancer activities. Extracts from seaweed were found to have a strong antioxidant effect [14,15] and Grateloupia polysaccharides could inhibit ECA-109 [16] and U87 cell proliferation [17].

To make the best use of effective polysaccharides and enhance their extraction yield, attention has been paid to the extraction methods of polysaccharides from plants and algae. Extraction methods developed include the ultrasound-, microwave- and enzyme-assisted extraction methods [18]. Considering its simple and economical conditions, hot water extraction [19] has been widely used as the classical method for polysaccharides extraction. However, hot water extraction involves a long time and high temperature, so the extraction method needs to be optimized.

Response surface methodology (RSM) is an effective statistical technique for optimizing complex processes. The main advantage is to reduce the experimental trials needed to evaluate multiple factors and their interactions. The Box-Behnken design (BBD), one type of RSM, is more efficient and easier to arrange and interpret results for than other methods [20]. It is widely used in optimizing extraction process variables, such as polysaccharides, protein, anthocyanins and phenolic compounds [21].

According to our preliminary research, we separated the ethanol extract, petroleum ether, ethyl acetate, *n*-butyl alcohol and aqueous fractions from GL and discovered antioxidant, antibacterial and antischistosomal bioactivities [8]. In this experiment, we studied the extraction of aqueous fractions, or polysaccharides. The extraction conditions of polysaccharides were optimized to increase the extraction yield. Furthermore, we evaluated the scavenging and anticancer activities of GL polysaccharides with the aim of exploitation for medicines and foods.

## 2. Results and Discussion

### 2.1. Single-Factor Experimental Analysis of G. livida (Harv.) Yamada (GL) Polysaccharide Extraction

**Figure 1:**
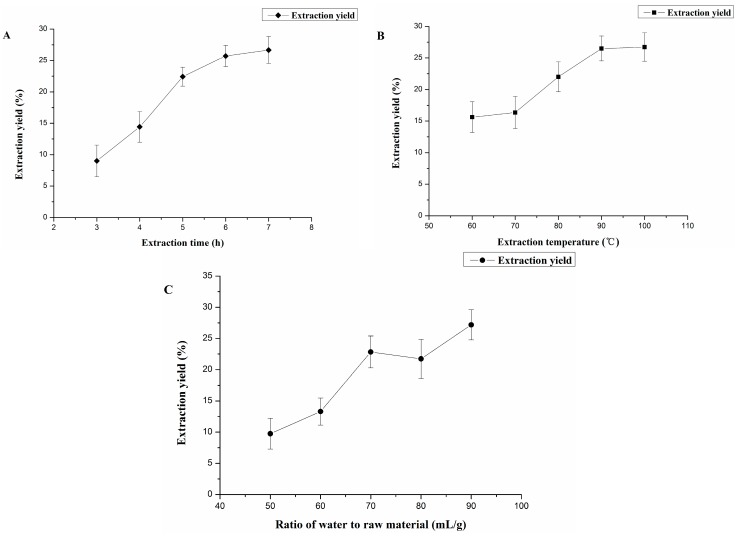
Effect of extraction time (**A**); extraction temperature (**B**); and ratio of water to raw material (**C**) on extraction yield of *G. livida* (Harv.) Yamada (GL) polysaccharides.

The extraction time, extraction temperature and ratio of water to raw material were studied and the results of single-factor experiments are shown in Figure 1. The three factors were effective for the yield of GL polysaccharides. The most suitable conditions were chosen for use in the BBD design.

#### 2.1.1. Effect of Extraction Time on Extraction Yield of GL Polysaccharides

The effect of extraction time on the yield of GL polysaccharides was investigated with extraction time 3 to 7 h (Figure 1A), with other extraction parameters fixed, such as an extraction temperature of 90 °C and ratio of water to raw material of 70 mL/g. The extraction yield of GL polysaccharides significantly increased from 9.02% to 22.42% with extraction time varying from 3 h to 5 h; it began to plateau with proceeding extraction. The polysaccharides need much time to swell so that they can be released and diffused into water [22]. However, extended extraction time may lead to the degradation of the polysaccharides [23]. Therefore, an extraction time ranging from 4 h to 6 h is the best for polysaccharide extraction.

#### 2.1.2. Effect of Extraction Temperature on Extraction Yield of GL Polysaccharides

The extraction yield of GL polysaccharides affected by extraction temperature is in Figure 1B. The extraction process was performed with temperatures from 60 °C to 100 °C, with the other extraction variables such as ratio of water to raw material and extraction time fixed at 70 mL/g and 5 h, respectively. The extraction yield of GL polysaccharides increased with increasing extraction temperature from 60 °C to 80 °C, then peaked at 26.73% ± 2.25% at 100 °C. With increasing extraction temperature, the polysaccharide diffusion coefficient increases accordingly, for enhanced solubility of polysaccharides in the extracting solvent [24]. We selected temperatures from 80 °C to 100 °C as the appropriate extraction temperature.

#### 2.1.3. Effect of Ratio of Water to Raw Material on Extraction Yield of GL Polysaccharides

Different ratios of water to raw material have an effect on extraction yield (Figure 1C). In this study, we used ratios of water to raw material from 50 mL/g to 90 mL/g, with other parameters (extraction time and extraction temperature) fixed at 0 level (5 h and 90 °C). Extraction yield increased with ratio of water to material from 50 mL/g to 70 mL/g and decreased before it peaked at 27.19% ± 2.41% at 90 mL/g. Under the appropriate condition of ratio of water to raw material, the polysaccharides can swell thoroughly, and more polysaccharide molecules could dissolve in water to improve extraction yield [22]. If ratio of water to raw material is too small, polysaccharides cannot be completely extracted, and if too high, the cost will increase [25]. Thus, we chose ratios of water to material from 60 mL/g to 80 mL/g as the optimal conditions.

According to the single-factor study, the following conditions could be used for the BBD (Box-Behnken design) experiments: extraction time of 4–6 h, extraction temperature of 80–100 °C and ratio of water to raw material of 60–80 mL/g.

### 2.2. Box-Behnken Design (BBD) and Analysis

#### 2.2.1. Statistical Analysis

Table 1 shows the BBD with three factors and three levels examined to optimize the mutual effect of three independent variables (extraction time, extraction temperature and ratio of water to material) on extraction yield of GL polysaccharides. The design matrix and experimental and predicted extraction yield of polysaccharides are in Table 2. The yield of GL polysaccharides ranged from 20.3% to 39.48% and the maximum yield was with ratio of water to raw material 70 mL/g at 100 °C and 4 h extraction time. On multiple regression analysis, the quadratic polynomial equation for the independent variables and response variable can be expressed as follows:

(1) $$\text{Y = 37.79 + 1.27}{\text{X}}_{1}\text{ + 1.55}{\text{X}}_{2}\text{ - 1.84}{\text{X}}_{3}\text{ - 1.22}X_{1}^{2}\text{ - 0.17}{\text{X}}_{2}^{2}\text{ - 7.65}{\text{X}}_{3}^{2}\text{ - 1.84}{\text{X}}_{1}{\text{X}}_{2}\text{ + 5.10}{\text{X}}_{1}{\text{X}}_{3}\text{ + 2.73}{\text{X}}_{2}{\text{X}}_{3}$$

**Table 1:** Factors and levels in the Box-Behnken design used to examine extraction yield of *G. livida* (Harv.) Yamada (GL) polysaccharides.

Independent Symbol Variables	Factor Level		
	−1	0	1
Extraction X_1_ (h) time	4	5	6
Extraction X_2_ (°C) temperature	80	90	100
Ratio of water X_3_ (mL/g) to raw material	60	70	80

**Table 2:** Design and results of Box-Behnken design used to examine extraction yield of *G. livida* (Harv.) Yamada (GL) polysaccharides.

Run	Factor Level			Extraction Yield (%)	Extraction Yield (%)	Residue (%)
	X_1_ (h)	X_2_ (°C)	X_3_ (mL/g)	Experimental	Predicted	
1	4	80	70	31.07	31.75	−0.68
2	4	100	70	39.48	38.53	0.95
3	6	80	70	37.01	37.96	−0.95
4	6	100	70	38.06	37.44	1.62
5	5	80	60	33.53	33.00	0.53
6	5	80	80	24.96	23.86	1.10
7	5	100	60	29.53	30.63	−1.10
8	5	100	80	31.90	32.43	−0.53
9	4	90	60	34.75	34.60	0.15
10	6	90	60	27.35	26.93	0.42
11	4	90	80	20.30	20.72	−0.42
12	6	90	80	33.31	34.40	−1.09
13	5	90	70	39.23	37.79	1.44
14	5	90	70	36.52	37.79	−1.27
15	5	90	70	37.63	37.79	−0.16

A summary of the ANOVA parameters for the fitted quadratic polynomial model for extraction yield of GL polysaccharides is in Table 3. The *F*-test was used to check the statistical significance of the regression equation and *p* values to check the significance of each coefficient, which in turn may indicate the pattern of the interactions between the variables [26]. The model *f*-value for the model was 24.01 and the *p*-value was 0.0014, so the model was significant. The probability that the large *f*-value could occur due to noise was 0.14%. The smaller the *p*-values, the larger the significance of the corresponding coefficient [27]. The linear coefficients (X_2_ and X_3_), interaction terms (X_1_X_2_, X_1_X_3_ and X_2_X_3_) and quadratic term (${\text{X}}_{3}^{2}$) were significant (*p* < 0.05) (Table 3). The adequacy of the model could be confirmed by the determination coefficient (*R*^2^) [28]. The determination coefficient (*R*^2^ = 0.9774) suggested that the model was valid, implying that 97.74% of the variation could be explained by the fitted model. The adjusted determination coefficient was used to evaluate the correlation between the experimental values and predicted values, and the outcome ($R_{Adj}^{2}$ = 0.9367) suggested that the correlation was significant. The *f*-value (6.12) for “the lack of fit” indicated that the “lack of fit” was not significant relative to the pure error (*p* > 0.05). As a general rule, the coefficient of variation (CV) should not be >10% [29]. The CV for yield of GL polysaccharides was 4.25%, which indicated a good reliability of the experimental values. Adequate precision measures the signal to noise ratio. Its desired value is ≥4 [30]. The adequate precision value for our model of 15.554 indicated an adequate signal and that the model could be used to navigate the design space.

**Table 3:** ANOVA of the experimental data.

Source	Sum of Squares	df	Mean Square	*f*-Value	*p* > *f*
Model	424.74	9	47.19	24.01	0.0014
X_1_	12.83	1	12.83	6.52	0.0510
X_2_	19.22	1	19.22	9.78	0.0261
X_3_	26.97	1	26.97	13.72	0.0139
X_1_X_2_	13.54	1	13.54	6.89	0.0468
X_1_X_3_	104.14	1	104.14	52.97	0.0008
X_2_X_3_	29.92	1	29.92	15.22	0.0114
${\text{X}}_{1}^{2}$	5.50	1	5.50	2.80	0.1553
${\text{X}}_{2}^{2}$	0.10	1	0.10	0.053	0.8271
${\text{X}}_{3}^{2}$	215.82	1	215.82	109.78	0.0001
Residual	9.83	5	1.97		
Lack of fit	6.12	3	2.04	1.10	0.5090
Pure error	3.71	2	1.86		
Total model	434.57	14			
	*R*^2^ = 0.9774	$R_{Adj}^{2}$ = 0.9367	CV = 4.25%	Adequate precision = 15.554	

*R*^2^, determination coefficient; $R_{Adj}^{2}$, adjusted *R*^2^; CV, coefficient of variation.

#### 2.2.2. Optimization of GL Polysaccharide Extraction

**Figure 2:**
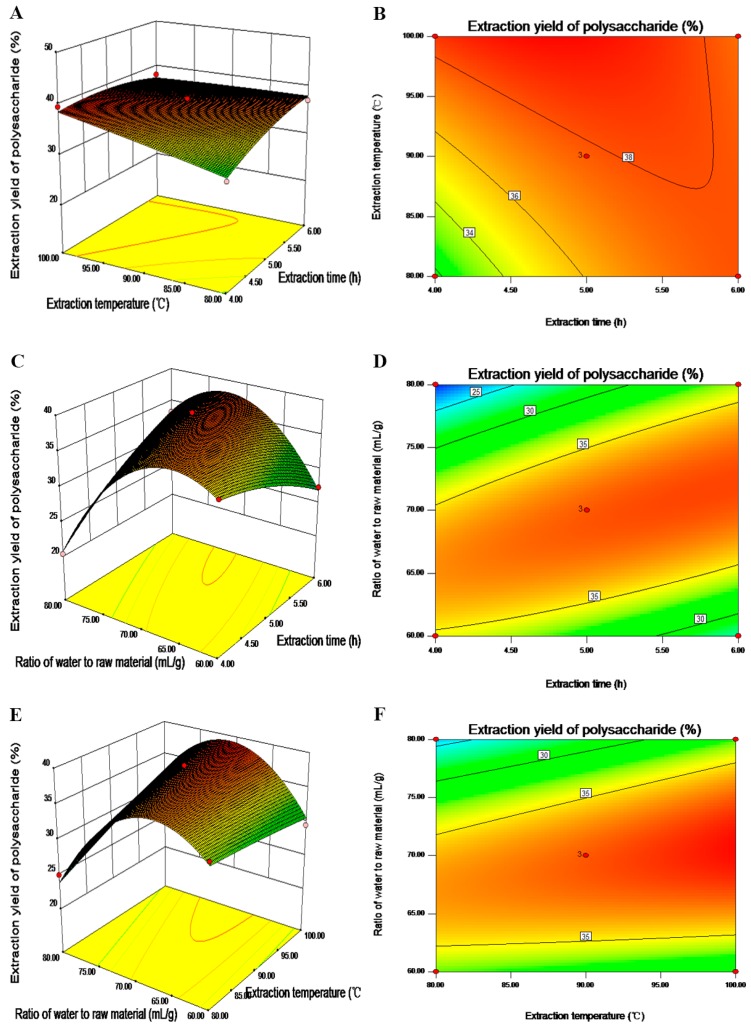
Response surface plots (**A**,**C**,**E**) and contour plots (**B**,**D**,**F**) showing the interactive effects of extraction time (X_1_), extraction temperature (X_2_) and ratio of water to raw material (X_3_) on yield of GL polysaccharides.

The response surface methodology was used to investigate the interactions of the variables and determine the optimal level of each variable for the maximal response [31]. The 3D response surface and 2D contour plots provided graphical representations of the regression equation (Figure 2), providing a means of visualizing the mutual effect of independent variables at different levels on the extraction yield of polysaccharides and the interactions between the independent variables. The 3D response surface plot demonstrated the mutual effect of two variables, with the other variable maintained at the respective zero level. Figure 2A depicts the interaction effect of extraction time (X_1_) and extraction temperature (X_2_) on the yield of GL polysaccharides with the ratio of water to raw material (X_3_) fixed at 70 mL/g. The yield of GL polysaccharides increased slightly with the increasing extraction time and temperature. Furthermore, the contour plot (Figure 2B) showed significant interactions between two variables (*p* < 0.05, Table 3). The extraction time slightly increased yield of GL polysaccharides, whereas yield increased rapidly with ratio of water to raw material from 60 mL/g to 68 mL/g (Figure 2C). However, with a further increase of the ratio of water to raw material, the yield decreased. Maximal extraction yield of GL polysaccharides could be achieved with extraction time and ratio of water to raw material 5 h and 70 mL/g, respectively. The contour plot (Figure 2D) and the results from Table 3 (*p* < 0.05) show significant reciprocal interactions between the two variables. The yield of GL polysaccharides increased with increasing ratio of water to raw material and after yield peaked, it decreased slowly (Figure 2E). However, yield increased little with increased extraction temperature when extraction time was set at 5 h. The relationships between the two variables were significant (*p* < 0.05, Table 3, Figure 2F). In conclusion, the highest yield of GL polysaccharides could be obtained with the extraction temperature and ratio of water to raw material of about 100 °C and 70 mL/g, respectively.

#### 2.2.3. Verification of Predictive Model

According to the Figure 2 and Equation (6), the optimal extraction conditions for GL polysaccharides were as follows: extraction time (X_1_) 4.63 h, extraction temperature (X_2_) 100 °C and ratio of water to raw material (X_3_) 69.35 mL/g. Under these optimal extraction conditions, the theoretical maximal extraction yield of GL polysaccharides was predicted to be 39.25% by the mathematical model. Considering operability in the experiment, the optimal conditions could be adjusted to the following conditions: extraction time (X_1_) 5 h, extraction temperature (X_2_) 100 °C and ratio of water to raw material (X_3_) 70 mL/g. To ensure the validation of the model equation, we performed three independent experiments under the modified polysaccharide extraction conditions. The mean polysaccharide extraction was 39.22% ± 0.09%, which agrees well with the predicted value for the good correlation between experimental and predicted values, and the response model is adequate for optimization.

### 2.3. Scavenging Activity

#### 2.3.1. DPPH Radical Scavenging Activity

**Figure 3:**
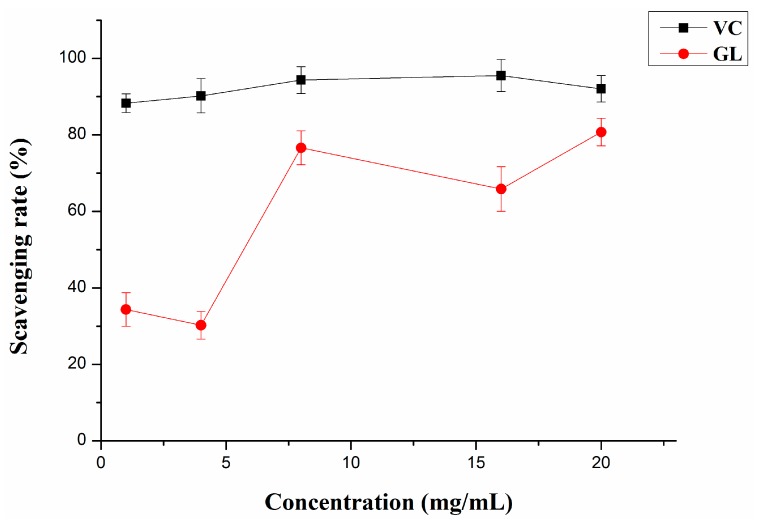
DPPH radical scavenging activity of GL polysaccharides and VC. Data are mean ± SD (*n* = 3).

The free radical scavenging activity of GL polysaccharides were measured by DPPH assay. DPPH, a stable *N*-centered free radical, has been often used to analyze the ability of free-radical scavenging properties or hydrogen donation of compounds and medicine materials [32]. As shown in Figure 3, DPPH scavenging by GL polysaccharides increased rapidly with increasing concentrations from 4 mg/mL to 8 mg/mL, and then grew steadily to peak at 80.71% with 20 mg/mL concentration. Therefore, GL polysaccharides, especially at high concentration, had a noticeable scavenging activity on DPPH radicals. Nevertheless, compared to the scavenging rate of the positive standard (VC; ascorbic acid), the antioxidant activity of GL polysaccharides was not as good as VC. The possible mechanism may be their electron donation power to free radicals, thereby terminating the radical chain reaction [33].

#### 2.3.2. Hydroxyl Radical Scavenging Activity

**Figure 4:**
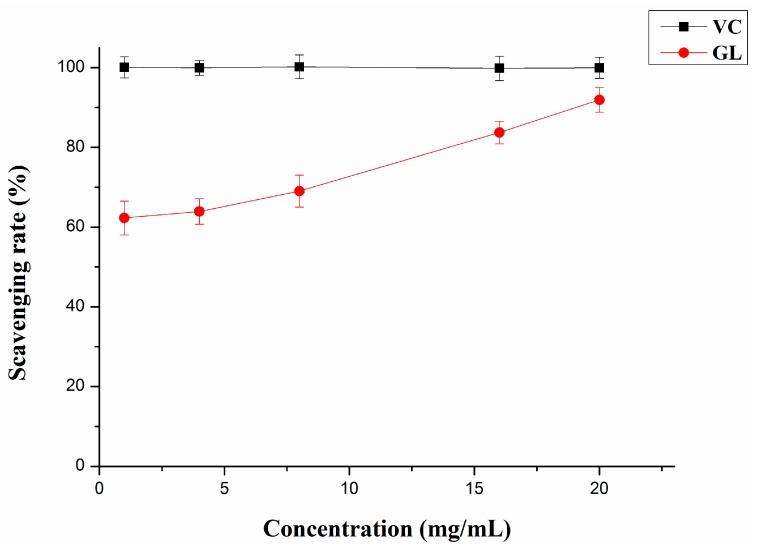
Hydroxyl radical scavenging activity of GL polysaccharides and VC. Data are mean ± SD (*n* = 3).

The hydroxyl radical is considered the most reactive and poisonous free radical and induces severe damage to adjacent biomolecules as an active initiator for lipids peroxidation [34]. The reaction system containing Fe^2+^-H_2_O_2_-salicylic acid in the aqueous phase was used to generate OH [35] to measure the scavenging activity of GL polysaccharides for hydroxyl radicals (Figure 4). With increasing concentration of GL polysaccharides, the hydroxyl radical scavenging activity increased, and at 20 mg/mL concentration, its scavenging rate peaked. Furthermore, the antioxidant activity of VC was greater than with GL polysaccharides. Previous studies have suggested that the antioxidant mechanism may be due to the supply of hydrogen by polysaccharides, which combine with radicals and form a stable radical to terminate the radical chain reaction [36]. GL polysaccharides may scavenge hydroxyl radicals by providing hydrogen. However, the exact mechanism relating to the hydroxyl radical scavenging activity by polysaccharides needs further study.

### 2.4. Anticancer Activity

**Figure 5:**
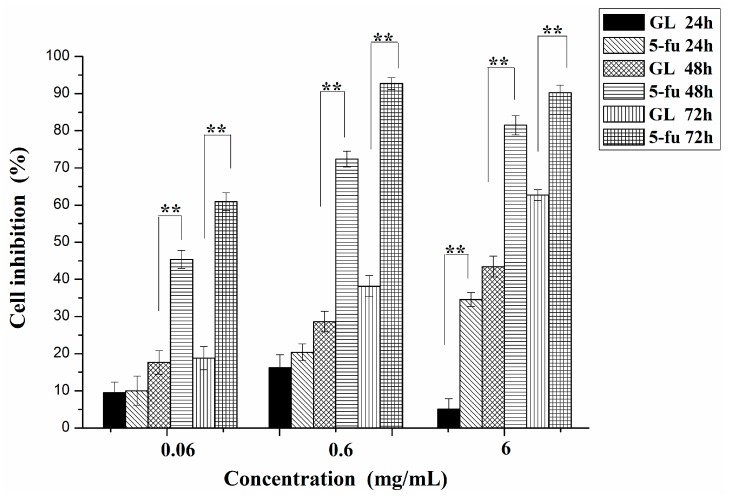
GL polysaccharides and 5-fu were incubated with HepG2 cells for 24, 48, and 72 h (*n* = 3). ** *p* ˂ 0.01.

We evaluated the cell inhibition rates of GL polysaccharides on HepG2 cells after incubation at three intervals (24 h, 48 h, and 72 h) via MTT assay. As shown in Figure 5, the growth of HepG2 cells was subject to different inhibition rates at different times with different concentrations of GL polysaccharides, representing the time-effect relationship. With increasing incubation time, the inhibition rate of HepG2 cells increased. The results were significant except for cells incubated between 48 h and 72 h at 0.06 mg/mL. Otherwise, the GL polysaccharides inhibited the proliferation of HepG2 cells dose-dependently. However, with 24 h incubation, cell growth was not inhibited significantly at a low concentration of GL polysaccharides, so the polysaccharides had no useful effects until they were at the suitable concentration. As a positive standard, 5-fluorouracil (5-fu) had a greater effect on HepG2 cells than GL polysaccharides did. However, incubation of GL polysaccharides and 5-fu at low concentrations (0.06l mg/mL and 0.6 mg/mL) were not significant (*p* ˃ 0.05). Oxidative stress is among the main causes of cancer-related death. If compounds can enhance the level of anti-oxidation and reduce reactive oxygen species content in cancer cells, they may inhibit cell growth [37]. Therefore, the anticancer activity of GL polysaccharides might be ascribed to its effect on scavenging free radicals.

## 3. Experimental Section

### 3.1. Plant and Cell Materials

*Grateloupia* *livida* (Harv.) Yamada (GL) was obtained from Nan Ao Island, Shantou, Guangdong Province, China, and identified by the Nan Ao Marine Biological Research Institute of Shantou University, China. The human liver carcinoma cell line HepG2 was obtained from the Medical College of Shantou University, China.

### 3.2. Chemicals and Reagents

2,2-Diphenyl-1-picrylhydazyl (DPPH), salicylic acid, ascorbic acid (VC), 3-[4,5-dimethylthiazole-2-yl]-2,5-diphenyltetrazolium bromide (MTT) and glucose were obtained from Sigma-Aldrich Chemical Co. (St. Louis, MO, USA). Other reagents, including ethanol, dimethyl sulfoxide (DMSO), phenol, concentrated sulfuric acid, hydrogen peroxide (H_2_O_2_), and ferrous sulfate (FeSO_4_) were from Shantou Xilong Chemical Factory (Shantou, China). Dulbecco’s modified eagle medium (DMEM) was from Thermo Fisher Scientific Inc. (Shanghai, China). Fetal bovine serum was from Biosun Science and Technology (Shanghai, China).

### 3.3. Preparation of GL

GL was washed, smashed, and dried at 60 °C to prepare the experimental sample. To remove lipid materials and other micromolecule matter thoroughly, GL powders were prepared by operating heating circumfluence with 95% ethanol, at 90 °C twice and 3 h each time. The purified GL powders were then filtrated and dried for use.

### 3.4. Polysaccharide Extraction from GL

The prepared GL powders (0.5 g) were extracted by water in a designed extraction time (3–7 h), extraction temperature (60–100 °C), and ratio of water to raw material (50–90 mL/g) and the extract liquid was concentrated in a rotary evaporator. The solution was precipitated by 80% ethanol and incubated for 12 h at 4 °C in the refrigerator before it was centrifuged at 3500 rpm for 15 min [38]. Then the precipitant was dissolved in water and frozen at −80 °C. Crude GL polysaccharides were obtained by lyophilization. The content of GL polysaccharides was measured by the common phenol-sulfuric acid method, with d-glucose used as the standard to construct a standard curve. The percentage polysaccharide yield (%) was calculated as follows [39]:

(2) $$GL\;polysaccharides\;extraction\;yield\;(\%)=\frac{\text{X × V}}{{\text{W × 10}}^{\text{3}}}$$

where X (mg/mL) is the concentration of GL polysaccharide solution, V (mL) is the volume of GL polysaccharide solution and W (g) is the dried powder of GL weight.

### 3.5. Single Factor Experimental Design

Single factor experiments were performed to investigate the effect of extraction time, extraction temperature, and ratio of water to raw material on GL polysaccharide extraction yield. During the optimization of experimental factors, one factor was changed and the other factors were kept constant in each experiment. All experiments were repeated three times.

### 3.6. BBD and Statistical Analysis

From the preliminary single factor experiment design, BBD was performed for further optimization of GL polysaccharide extraction. Three parameters, including extraction time, extraction temperature, and ratio of water to raw material, were designated X_1_, X_2_, and X_3_, respectively (Table 1). Each of the independent variables had coded levels of −1 (low), 0 (central), +1 (high).The quadratic polynomial equation reflecting the relationship between three independent variables and the response variable (extraction yield of GL polysaccharides) is as follows [40]:

(3) $${\text{Y = β}}_{0}\text{ + }\overset{3}{\underset{i=1}{\sum }}\beta_{\text{i}}{\text{X}}_{i}\text{ + }\overset{3}{\underset{i=1}{\sum }}{\text{β}}_{ii}{\text{X}}_{i}^{2}\text{ + }\overset{2}{\underset{i=1}{\sum }}\overset{3}{\underset{j=i+1}{\sum }}{\text{β}}_{ij}{\text{X}}_{i}{\text{X}}_{j}$$

where Y is the response variable; β_0_, β*_i_*, β*_ii_*, and β*_ij_* are regression coefficients of variables for intercept, linearity, quadratic, and interaction terms, respectively; and X*_i_* and X*_j_* are the coded independent variables (*i* ≠ *j*). In the whole design, 15 experimental runs were performed in random order, with 12 factorial runs and three center runs. The factorial point is the 3D vertex consisting of independent variables (X_1_, X_2_ and X_3_). Three experiments were performed at the center point (0) to evaluate the pure error [41]. The design of the experiments is in Table 2. All trials were performed in triplicate. Statistical analysis of data was performed to establish the mathematical model by the BBD method, evaluating the effects of each independent variable on the response.

### 3.7. Antioxidant Activity Assay in Vitro

#### 3.7.1. DPPH Radical Scavenging Activity

The DPPH radical scavenging activity assay was performed as described [42], with a few modifications. Briefly, 250 μL of 0.1 mM freshly prepared DPPH solution (in 95% ethanol) was added to 50 μL GL polysaccharide solution at 1 mg/mL, 4 mg/mL, 8 mg/mL, 16 mg/mL and 20 mg/mL, respectively. The mixture was shaken vigorously and incubated at 25 °C for 30 min in the dark, before measuring the absorbance at 517 nm. VC (ascorbic acid) was used as the positive standard. The GL polysaccharide ability to scavenge DPPH was calculated by the following equation:

(4) $$Scavenging\;rate\;(\%)=\frac{\text{1 }-{\text{ (A}}_{\text{i}}\;-{\text{ A}}_{\text{j}})}{{\text{A}}_{0}}\text{ × 100\%}$$

where A_i_ is the absorbance obtained from GL polysaccharides mixed with DPPH solution, A_j_ is the absorbance of GL polysaccharides with 95% ethanol and A_0_ is the absorbance of DPPH solution with 95% ethanol. Data are expressed as mean ± SD of three independent experiments.

#### 3.7.2. Hydroxyl Radical Scavenging Assay

The hydroxyl radical scavenging ability of GL polysaccharides was analyzed by the hydroxyl radical system generated by the Fenton reaction [43] with minor changes. In brief, GL polysaccharides were dissolved in distilled water at 1 mg/mL, 4 mg/mL, 8 mg/mL, 16 mg/mL and 20 mg/mL. The reaction mixture contained 50 μL FeSO_4_ (6 mM), 50 μL H_2_O_2_ (6 mM) and 100 μL GL polysaccharides of varying concentrations. After shaking and incubation at room temperature for 10 min, 50 μL salicylic acid was added to the mixture, then shaken and incubated at room temperature for another 30 min, and the absorbance of the mixture was measured at 510 nm. VC was considered the positive standard. The hydroxyl radical scavenging rate was calculated by the following equation:

(5) $$Scavenging\;rate\;(\%)=\frac{\text{1 }-{\text{ (A}}_{\text{i}}\;-{\text{ A}}_{\text{j}})}{{\text{A}}_{\text{0}}}\text{ × 100\%}$$

where A_i_ is the absorbance obtained from the reaction mixture (FeSO_4_ and H_2_O_2_) with GL polysaccharides and salicylic acid, A_j_ is the absorbance of the reaction mixture with GL polysaccharides and A_0_ is the absorbance of reaction mixture with salicylic acid. Data are expressed as mean ± SD of three independent experiments.

### 3.8. Cell Cytotoxicity Assay in Vitro

#### 3.8.1. Cell Culture

DMEM medium was supplemented with 10% fetal bovine serum, penicillin (100 units/mL) and streptomycin (100 units/mL). HepG2Cells were cultured in DMEM medium and incubated at 37 °C in a humidified 5% CO_2_ incubator.

#### 3.8.2. MTT Assay

The cytotoxic effect of GL polysaccharides on HepG2 cells was evaluated by the standard MTT assay. HepG2 cells were seeded at 5 × 10^4^ per well in 96-well microplates and incubated for 24 h to allow cell attachment. Then different concentrations of GL polysaccharides dissolved (0.06 mg/mL, 0.6 mg/mL, 6 mg/mL) in DMEM were added to each well in the plates. 5-fluorouracil (5-fu) was considered the positive standard. The cells cultured with DMEM medium were the control. The plates were incubated in a humidified 5% CO_2_ incubator at 37 °C for 24 h, 48 h, and 72 h, respectively. Then, 20 μL (5 mg/mL) MTT solution was added to the medium and further incubated for 4 h in the dark. The medium in each well was removed and 150 μL DMSO was added to dissolve the purple formazan crystals. The absorbance at 490 nm (A_490_) was read on microplate reader and the cell cytotoxicity rate was calculated as follows:

(6) $$Cell\;cytotoxicity\;rate\;(\%)=\frac{\text{1 }-{\text{A}}_{\text{i}}}{{\text{A}}_{\text{j}}}\text{ × 100\%}$$

where A_i_ is the absorbance from the treatment group and A_j_ is the absorbance of the control group. Data are expressed as mean ± SD from three independent experiments.

## 4. Conclusions

In summary, the polysaccharides from *G. livida* were extracted by the heating circumfluence method and the extraction process was successfully optimized by the BBD method. From the single-factor experiments, the optimization of the extraction conditions, including extraction time, extraction temperature and ratio of water to material, were determined as extraction time 5 h, extraction temperature 100 °C and ratio of water to raw material 70 mL/g. Under these conditions, the experimental yield of polysaccharides was 39.22% ± 0.09%, close to the predicted yield of 39.25%. Hence, the model established by BBD was adequate for GL polysaccharide extraction. The antioxidant and anticancer activities of GL polysaccharides were evaluated. GL polysaccharides possessed certain inhibitory effects on DPPH radical and hydroxyl radicals. For anticancer activity, GL polysaccharides can suppress HepG2 cell proliferation somewhat, but are not as good as 5-fu. The results obtained in this experiment should be useful for the further exploitation of the seaweed.
